# Electrochemistry Study of Permselectivity and Interfacial Electron Transfers of a Branch-Tailed Fluorosurfactant Self-Assembled Monolayer on Gold

**DOI:** 10.3390/molecules23112998

**Published:** 2018-11-16

**Authors:** Shanshan Li, Qingying Luo, Zhiqing Zhang, Guanghui Shen, Hejun Wu, Anjun Chen, Xingyan Liu, Meiliang Li, Aidong Zhang

**Affiliations:** 1 College of Food Science, Sichuan Agricultural University, Ya’an 625014, China; cherry12112009@163.com (Q.L.); zqzhang721@163.com (Z.Z.); shenghuishen@163.com (G.S.); hejunwu520@163.com (H.W.); anjunc003@163.com (A.C.); lxy05@126.com (X.L.); liml@sicau.edu.cn (M.L.); 2 Key Laboratory of Pesticide & Chemical Biology of Ministry of Education, College of Chemistry, Central China Normal University, Wuhan 430079, China

**Keywords:** branch-tailed fluorosurfactant, self-assembled monolayer, amphiphilic surface, permselectivity, electron transfer, “click” reaction

## Abstract

We investigated the permselectivity and interfacial electron transfers of an amphiphilic branch-tailed fluorosurfactant self-assembled monolayer (FS-SAM) on a gold electrode by cyclic voltammetry (CV) and electrochemical impedance spectroscopy (EIS). The FS-SAM was prepared by a self-assembly technique and a “click” reaction. The barrier property and interfacial electron transfers of the FS-SAM were also evaluated using various probes with different features. The FS-SAM allowed a higher degree of permeation by small hydrophilic (Cl^−^ and F^−^) electrolyte ions than large hydrophobic (ClO_4_^−^ and PF_6_^−^) ones. Meanwhile, the redox reaction of the Fe(CN)_6_^3−^ couple was nearly completely blocked by the FS-SAM, whereas the electron transfer of Ru(NH_3_)_6_^3+^ was easier than that of Fe(CN)_6_^3−^, which may be due to the underlying tunneling mechanism. For hydrophobic dopamine, the hydrophobic bonding between the FS-SAM exterior fluoroalkyl moieties and the hydrophobic probes, as well as the hydration resistance from the interior hydration shell around the oligo (ethylene glycol) moieties, hindered the transport of hydrophobic probes into the FS-SAM. These results may have profound implications for understanding the permselectivity and electron transfers of amphiphilic surfaces consisting of molecules containing aromatic groups and branch-tailed fluorosurfactants in their structures.

## 1. Introduction

Fluorosurfactants (FSs) are synthetic organofluorine chemical compounds that possess multiple fluorine atoms. Their molecular structure displays a fluorinated hydrophobic “tail” and a hydrophilic “head”, wherein the hydrophobic part consists of a fluorocarbon group and the hydrophilic part consists of a polyoxyethylene chain [1]. FSs are well known for their unique physicochemical properties, such as high surface activity, excellent high temperature and chemical resistance, as well as hydrophobic and oleophobic properties, that provides their high suitability for various field applications [2,3]. For example, FSs are widely used to change the hydrophobicity of surfaces in applications concerning antifogging [4], antimicrobial [5], oil-water separation [6], and preparation of electrodes [7] and sensors [8]. Consequently, properties regarding FS-modified surfaces have attracted increasing attention and have been widely investigated in different fields, especially in the electrochemical field [9,10,11].

Great efforts have been devoted to electrochemical studies for in-depth understanding of fundamental structure–property relationships of FS-modified surfaces. For instance, Chen et al. [12,13] investigated applications of FS-modified surfaces in electroanalysis. They found that the electro-oxidation of l-cysteine and tertiary amines becomes more facile at the FS-modified gold electrode. However, most of the reports focused on straight-chain FS-modified surfaces. No direct observations of interfacial phenomena of branch-tailed FS-modified surfaces have been reported. Strengthening the research on branch-tailed FS-modified surfaces and the correlation between molecular structure and surface properties, especially in foundational electrochemical fields, is thus needed.

**Scheme 1:**
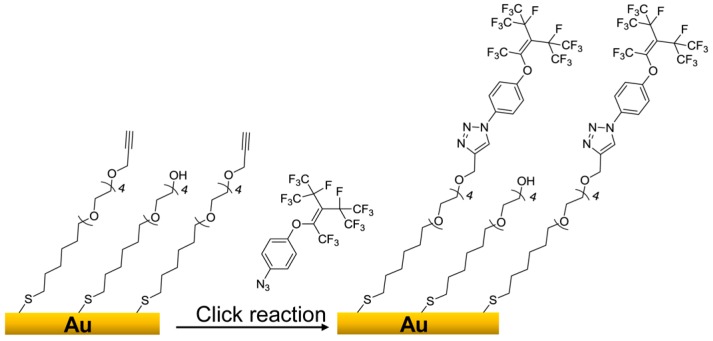
Schematic illustration of the fabrication of the FS-SAM on gold.

As an attractive way to functionalize solid surfaces, self-assembled monolayer (SAM) technology offers a unique tool for constructing well-defined, stable structures on surfaces with controlled physicochemical characteristics for a variety of applications [14,15]. Most of the abovementioned investigations are based on the SAM technology. The “click” reaction is one of the most versatile and beneficial chemistry tools in surface modifications [16]. Previous reports showed that this chemoselective and site-specific modification technique can be implemented in the fabrication of SAM-modified electrodes via the triazole-forming “click” reaction between azide-terminated SAM-modified electrodes and alkynyl-terminated molecules [17,18]. In this work, a branch-tailed FS-SAM has been prepared on a gold electrode surface by using the SAM technology and the “click” reaction, as shown in Scheme 1, and was used in a comparative study of various supporting electrolyte ion penetration in the monolayer. Furthermore, the effect of the FS-SAM system on the electrochemical behavior of various probes with different features was also studied.

For fabricating the FS-SAM on gold surface, we first mixed ω-tetra (ethylene glycol) hexanethiol (HS(CH_2_)_6_(OCH_2_CH_2_)_4_OCH_2_-C≡CH, OEG-CCH) and ω-propargyl tetra (ethylene glycol) hexanethiol (HO(CH_2_CH_2_O)_4_(CH_2_)_6_SH, OEG-OH). The obtained mixed thiols were then self-assembled onto the surface of the gold electrode. Subsequently, azide-derivatized *p*-perfluorononenyloxy benzene (N_3_-Ar-O-C_9_F_17_) was linked to the propargyl-terminated OEG-CCH by forming a stable 1,2,3-triazole ring via a copper(I)-catalyzed “click” reaction. A branch-tailed FS-SAM was then formed on the gold electrode surface. Various techniques, such as X-ray photoelectron spectrometry (XPS), grazing incidence reflectance (GIR) infrared spectroscopy, and contact angle (CA) measurements, were used to characterize the prepared surfaces. Cyclic voltammetry (CV) and electrochemical impedance spectroscopy (EIS) measurements were performed to evaluate the barrier property of the FS-SAM using Fe(CN)_6_^3−^, Ru(NH_3_)_6_^3+^, and dopamine as redox probes in the presence of different electrolyte ions. We focus primarily on the effect of film structure on the electrolyte ion penetration and the interfacial electron transfers of structurally different probes. These studies provide a background for future application of FSs in electrochemical systems.

## 2. Results

### 2.1. Characterization of the FS-SAM

**Figure 1:**
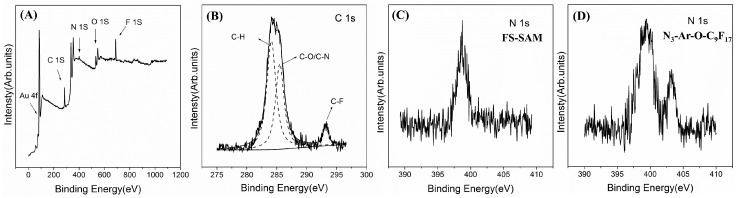
X-ray photoelectron spectrometry (XPS) wide-scan spectrum (**A**); C 1s core-level spectrum (**B**); and N 1s core-level spectrum (**C**) of the fluorosurfactant self-assembled monolayer (FS-SAM); and XPS N 1s core-level spectrum of N_3_-Ar-O-C_9_F_17_ (**D**).

The grafting of N_3_-Ar-O-C_9_F_17_ onto the SAMs was characterized by XPS, GIR spectroscopy, and CA measurement. The wide-scan XPS spectrum, as shown in Figure 1A, of the FS-SAM shows characteristic peaks from N 1s (399.9 eV) [19] and F 1s (687.9 eV) [20], which indicates the successful formation of triazole on the SAM. The C 1s core-level spectrum of the FS-SAM can be curve fit into three peak components with BEs of approximately 284.7, 286.4, and 293.1 eV, as shown in Figure 1B, attributable to the C–H, C–N/C–O [21], and C–F [20], respectively. The XPS N 1s results in Figure 1C,D supply additional evidence of triazole formation on FS-SAM. For the FS-SAM, only a single peak was observed at 399.8 eV, as shown in Figure 1C, whereas the control N 1s spectrum of N_3_-Ar-O-C_9_F_17_ shows two N 1s peaks at 399.8 and 403.6 eV, as shown in Figure 1D, respectively. The splitting of N 1s peak indicates the presence of two N species in N_3_-Ar-O-C_9_F_17_, which reflects the different charged state of N atoms in the azide group [22]. The single peak at 399.8 eV for the FS-SAM confirms the reaction between the azide and propargyl groups.

**Figure 2:**
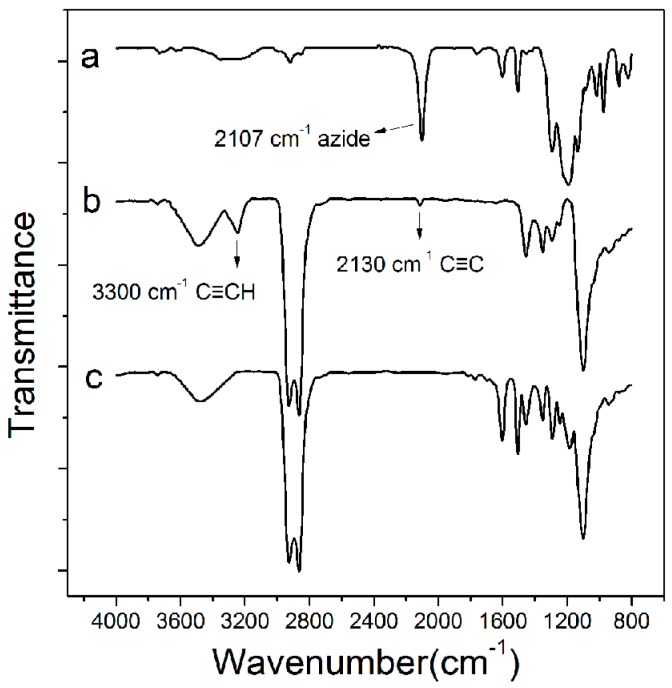
Grazing incidence reflectance (GIR) spectra of the N_3_-Ar-O-C_9_F_17_ (curve **a**), the binary OEG-SAM (curve **b**), and the FS-SAM (curve **c**).

In addition, GIR spectra, as shown in Figure 2, also suggest that “click” chemistry was successfully applied to the hydroxyl/propargyl-terminated binary OEG-SAM. The GIR spectrum of N_3_-Ar-O-C_9_F_17_ (curve a) clearly reveals the appearance of an absorbance peak at 2107 cm^−1^, which is characteristic of the azide groups. The peaks at 1284, 1183, and 1135 cm^−1^ are attributed to C-F stretching vibrations [23] and peaks at 1600 and 1500 cm^−1^ are the characteristic stretching peaks of the C=C bond in the benzene ring [24]. The hydroxyl/propargyl-terminated binary OEG-SAM (curve b) shows characteristic peaks at 3300 and 2130 cm^−1^ (≡C-H and C≡C stretching) [25], 3500 cm^−1^ (-OH stretching), and 1103 cm^−1^ (C-O-C stretching) [24]. After the triazole formation with N_3_-Ar-O-C_9_F_17_, the peaks from the propargyl and azide groups disappeared and the characteristic C-F and benzene ring stretching peaks were observed on FS-SAM (curve c).

Moreover, CA measurements were used to characterize the surfaces. Initially, the water CA of the hydroxyl/propargyl-terminated SAM was 53 ± 2°, indicating a moderate wettability of the surface. After the N_3_-Ar-O-C_9_F_17_ grafting onto the surface, the CA value increased to 121 ± 2° for the formative FS-SAM, resulting from the highly hydrophobic -CF_3_ groups. All the above evidence demonstrates the successful grafting of N_3_-Ar-O-C_9_F_17_ into the hydroxyl/propargyl-terminated SAMs.

We further optimized the condition for preparing the FS-SAM, and characterized the composition of the monolayers by CA [26,27], EIS [28], and XPS measurements. The results revealed that the optimal assembly solution for preparing the binary OEG-SAM is with the OEG-CCH fraction of 25%. Under the optimization condition, the obtained FS-SAM consists of 56% FS and 44% OEG-OH. The details can be seen in Section 1 in the Supplementary material. Since the molecular chain of FS is longer than that of OEG-CCH, and the tailed -C_9_F_17_ group has a high occupied surface area, a compact and highly fluorinated surface can be obtained.

### 2.2. Electrolyte Ion Permeability of the FS-SAM

**Figure 3:**
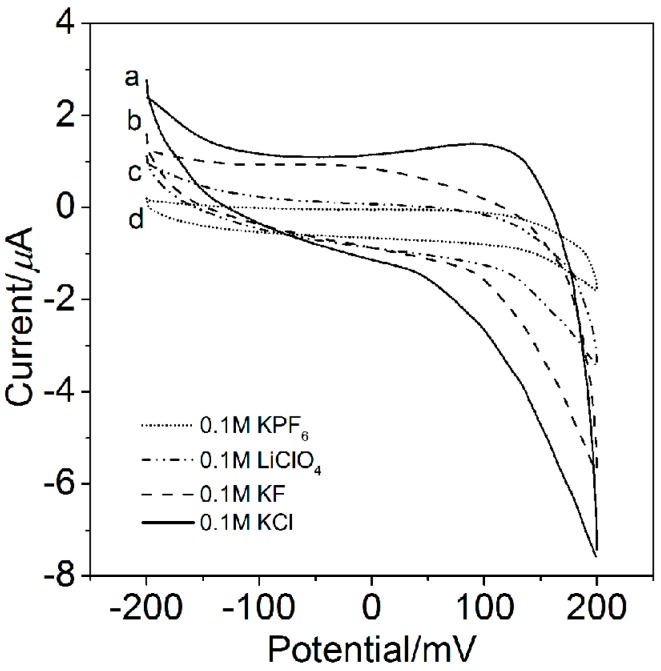
Cyclic voltammetry (CV) on the FS-SAM-modified gold electrode between −200 and 200 mV in different electrolyte solutions: 0.1 M KCl (curve **a**), 0.1 M KF (curve **b**), 0.1 M LiClO_4_ (curve **c**), and KPF_6_ (curve **d**). Scan rate, 50 mV/s. pH: 7.0.

The electrochemical response of a thin film is strongly influenced by electrolyte properties, such as ion charge, size, and hydrophilicity/hydrophobicity. It is well known that SAMs formed on substrates are not perfectly uniform but may have some defects and allow some degree of penetration by solvent molecules and electrolyte ions. To obtain insight into the ion permeability of the FS-SAM, we used CV to study the film capacitance (*C*) (*C* gives rise to current during the charging of the electrode double layer and exhibits a direct correlation with supporting electrolyte) in different inert electrolytes without any redox species. Figure 3 shows the CV data of the FS-SAM-modified gold electrode between −200 and 200 mV in 0.1 M KCl (curve a), 0.1 M KF (curve b), 0.1 M LiClO_4_ (curve c), and KPF_6_ (curve d) at 50 mV/s. Table 1 exhibits the calculated film capacitances from the corresponding CV data according to the formula *C* = *i_c_*/2*vA*, where *i_c_* is the charging current (μA), *v* is the scan rate (mV/s), and *A* is the electrode area (cm^2^) [29].

As shown in Table 1, the capacitance of the FS-SAM is larger in hydrophilic Cl^−^ [30] and F^−^ [31] solution than that in hydrophobic ClO_4_^−^ [32] and PF_6_^−^ [30] solution. The largest and the least film capacitance was obtained in the Cl^−^ and PF_6_^−^ solutions, respectively. The results indicate that the FS-SAM permits the solvent and the hydrophilic ions, but not the hydrophobic ions, to permeate into it. On one hand, this phenomenon might be ascribed to the hydrophobic bond formation between the hydrophobic ions and the *p*-perfluorononenyloxy benzene moieties at the exterior of the FS-SAM. On the other hand, amphiphilic SAMs that consists of OEG and hydrophobic components have been reported to possess a hydration shell around the OEG moieties [33]. Transporting into the hydration interior of the FS-SAM is thermodynamically unfavorable for the hydrophobic ClO_4_^−^ and PF_6_^−^ ions. Consequently, the capacitance of the FS-SAM decreased in hydrophobic electrolyte solutions.

Moreover, the ionic sizes of ClO_4_^−^ and PF_6_^−^ ions are larger than the sizes of Cl^−^ and F^−^. The large sizes of the former will certainly defy their chances of permeation. Meanwhile, we observed that the capacitance of the FS-SAM is smaller in KF than that in KC1 solutions. This finding means that FS-SAM allows C1^−^ to permeate into the interior more easily compared with F^−^, which exhibits good hydrophilicity. This result may also be ascribed to the large size of the hydrated F^−^, thus hindering its transport in the layer. A similar phenomenon about the film capacitance of the alkanethiol SAMs in C1^−^ and F^−^ solutions has been observed by Porter and coworkers [34].

### 2.3. Electrochemical Response of Hydrophilic Probes on the FS-SAM

CV and EIS are powerful tools to investigate the electrochemical properties of SAM-modified electrodes by using redox probes. In this study, two hydrophilic redox probes, namely, Fe(CN)_6_^3−^ and Ru(NH_3_)_6_^3+^ [35], were used to investigate the blocking property and interfacial electron transfers of the FS-SAM. The control experiments on the binary OEG-SAM-modified gold electrode and bare gold are also performed for comparison. 

#### 2.3.1. Fe(CN)_6_^3−^ Redox Reaction

**Figure 4:**
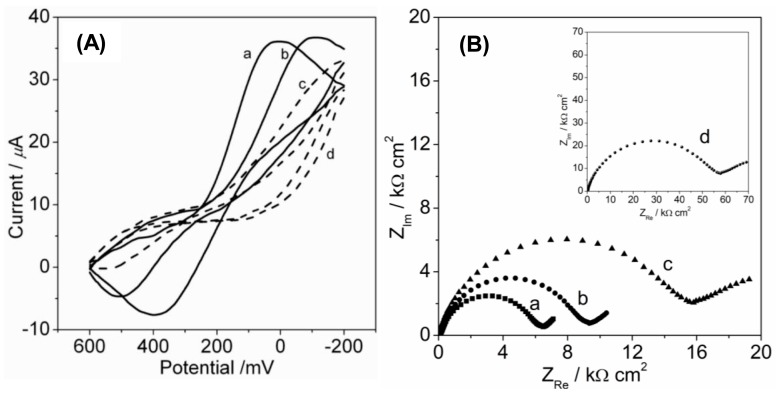
CV (**A**) and electrochemical impedance spectroscopy (EIS) (**B**) of 10 mM Fe (CN)_6_^3−^ in different electrolyte solutions on the binary OEG-SAM and the FS-SAM-modified gold electrodes: the binary OEG-SAM-modified gold electrodes in 0.1 M KCl (curve **a**) and 0.1 M KPF_6_ (curve **b**) and the FS-SAM-modified gold electrodes in 0.1 M KCl (curve **c**) and 0.1 M KPF_6_ (curve **d**). CV scan rate, 100 mV/s; pH: 7.0.

Figure 4A shows the CV data and Figure 4B shows the EIS data of the SAM-modified gold electrodes in 10 mM K_3_Fe(CN)_6_ with different supporting electrolytes. Each of the EIS spectra consists of a semicircle portion and a linear line portion, referring to the electron transfer reaction and diffusion, respectively. The semicircle diameter represents the charge transfer resistance (*R_ct_*) at the electrode surface. Using the corresponding *R_ct_* values from the EIS spectra, the rate constant (*k*_0_) values of Fe(CN)_6_^3−^ for the binary OEG-SAM and FS-SAM-modified electrodes were determined (details can be seen in Section 2 in the Supplementary Information). The obtained results of CV and EIS analysis are summarized in Table 2.

The peak potential difference (Δ*E_P_*) and *R_ct_* values are used as a measure of electron transfer rate on the SAM-modified electrodes, with larger Δ*E_P_* and *R_ct_* indicating a slower electron transfer, and we interpret any increase in this value to be caused by surface imperfections or barriers. On the bare gold electrode (data not shown), Fe(CN)_6_^3−^ displays a well-defined response in 0.1 M KCl solution, with a CV Δ*E_p_* value of 72 mV and an *R_ct_* value of 0.96 Ω cm^2^. Figure 4A,B and Table 2 show that the Δ*E_p_* and *R_ct_* values (curve a) of the binary OEG-SAM-modified electrodes in 0.1 M KCl solution, as expected, are high owing to the inhibited electron transfer by the OEG-SAM on the electrode surface. Additionally, the increase in Δ*E_p_* and *R_ct_* was great when KPF_6_ (curve b) was used as an electrolyte instead of KCl (curve a). This result indicates that the electron transfer rate between the electrode and Fe(CN)_6_^3−^ is higher for Cl^−^ compared with PF_6_^−^, with a *k*_0_ value of 0.42 and 0.28 cm·s^−1^, respectively. The variation in the electron transfer rate with a change in the anion can be explained by the hydrophobicity of the counterion. Given that the chloride ion is less hydrophobic, its migration in and out of the hydrophilic OEG monolayer is more favorable compared with that of PF_6_^−^.

After the FS-SAM formation, CV analysis revealed that the redox reaction of Fe (CN)_6_^3−^ is further inhibited by the FS monolayer, as shown in Figure 4A, curves c and d. Figure 4B shows the comparison of the impedance plots of the binary OEG-SAM and FS-SAM-modified electrodes in 10 mM K_3_Fe(CN)_6_ with KCl or KPF_6_ as the supporting electrolyte. The impedance plots of the FS-SAM, as shown in Figure 4B, curves c and d, show large semicircles in the high frequency region, suggesting a higher *R_ct_* and a stronger blocking ability of the FS-SAM compared with the binary OEG-SAM. Apparently, the electron transfer kinetics of Fe(CN)_6_^3−^ decreased in all cases for the FS-SAM systems compared with the binary OEG-SAM systems. Meanwhile, the electron transfer kinetics was lower when KPF_6_ was used as an electrolyte compared with KCl, *k*_0_ values are 0.17 and 0.05 cm·s^−1^ in the presence of PF_6_^−^ and Cl^−^ ions, respectively. The results are consistent with our previous hypothesis based on the capacitance of the FS-SAMs. The hydrophobic binding of negatively charged PF_6_^−^ ions to the hydrophobic *p*-perfluorononenyloxy benzene moieties creates an additional energetic barrier for the transport of negative Fe(CN)_6_^3−^ in the monolayer and thus hinders the faradaic reactions.

#### 2.3.2. Ru(NH_3_)_6_^3+^ Redox Reaction

**Figure 5:**
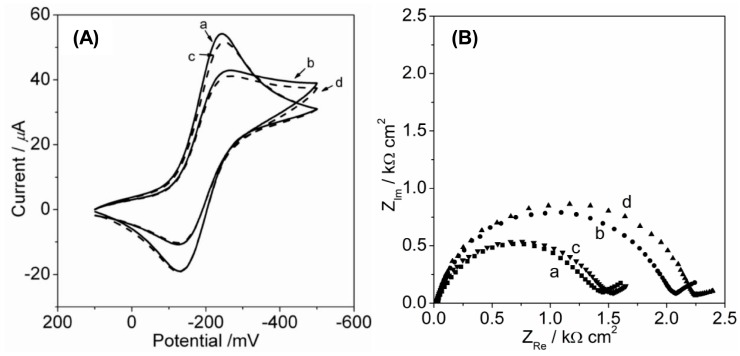
CV (**A**) and EIS (**B**) of 10 mM Ru(NH_3_)_6_^3+^ in different electrolyte solutions on the binary OEG-SAM and the FS-SAM-modified gold electrodes: the binary OEG-SAM-modified gold electrodes in 0.1 M KCl (curve **a**) and 0.1 M KPF_6_ (curve **b**), and the FS-SAM-modified gold electrodes in 0.1 M KCl (curve **c**) and 0.1 M KPF_6_ (curve **d**). CV scan rate, 100 mV/s. pH: 7.0.

Figure 5A shows the CV plots and Figure 5B shows the impedance plots of the SAM-modified gold electrodes in 10 mM Ru(NH_3_)_6_Cl_3_ with different supporting electrolytes. Table 3 summarizes the obtained results of CV and EIS analysis. On the bare gold electrode (data not shown), for Ru (NH_3_)_6_^3+^ in the presence of 0.1 M KCl, Δ*E_p_* (83 mV) and *R_ct_* (1.24 Ω cm^2^) values are comparable with those for Fe(CN)_6_^3−^ obtained under the same condition, indicating that fast kinetics can be obtained for both the probes on the bare gold electrode.

Unexpectedly, on the binary OEG-SAM-modified electrode, the CV of Ru(NH_3_)_6_^3+^ in the presence of 0.1 M KCl, as shown in Figure 5A, shows that the Ru(III) redox reaction is facile. The small Δ*E_p_* (114 mV) implies that the reaction is quasireversible. Such a behavior indicates that the binary OEG-SAM shows a poor blocking ability to the redox reaction of Ru(NH_3_)_6_^3+^, which is in contrast to that for Fe(CN)_6_^3−^. When KPF_6_ was used as an electrolyte, both Δ*E_p_* and *R_ct_* increased, which are in conformity with our observations using Fe(CN)_6_^3^ discussed previously. The hydrophobicity of PF_6_^−^ may hinder its migration in and out of the hydrophilic OEG monolayer.

The significantly different apparent kinetics of Ru(NH_3_)_6_^3+^ and Fe(CN)_6_^3−^ on the binary OEG-SAM needs to be clarified. Compared with Fe(CN)_6_^3−^, the small Δ*E_p_* and *R_ct_* values of Ru(NH_3_)_6_^3+^ indicate that the latter displays much faster kinetics than the former. The electrostatic interaction at the monolayer/solution interface may play an important role in the observed response. The binary OEG-SAMs can be assumed to be negatively charged in aqueous solution due to tightly bound hydroxide ions with a hydration shell, similar to the results of OEG- and methyl-terminated SAMs [36,37]. The negative hydration shell may be favorable for the positively charged probe Ru(NH_3_)_6_^3+^ to be accessed.

**Scheme 2:**
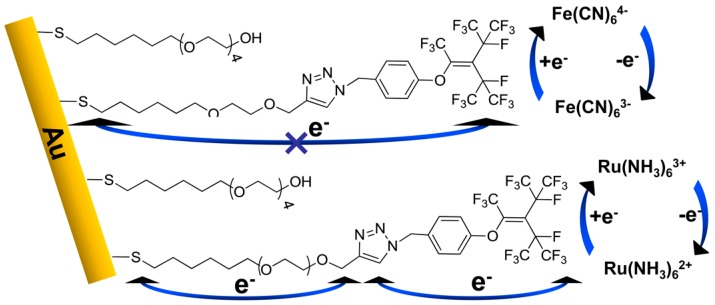
Proposed electron transfer mechanism for Fe(CN)_6_^3−^ and Ru(NH_3_)_6_^3+^ on the FS-SAM-modified electrode.

Interestingly, the expected blocking behavior for Fe(CN)_6_^3−^ on the FS-SAM was not observed for Ru(NH_3_)_6_^3+^. The response of Ru(NH_3_)_6_^3+^ on the FS-SAM, in fact, is less suppressed compared with that on the binary OEG-SAM. Fe(CN)_6_^3−^ (6 Å) and Ru(NH_3_)_6_^3+^ (6.4 Å) have comparable sizes, we conclude, if the redox reaction of Ru(NH_3_)_6_^3+^ is assumed to occur by access to the electrode surface through the pinhole defects present in the SAMs, the redox reaction of Fe(CN)_6_^3−^ should also exhibit facile kinetics. Given that this phenomenon was not observed, the Ru(NH_3_)_6_^3+^ redox reaction does not occur by access of the redox species to the electrode surface but by electron tunneling through the bonds, as shown in Scheme 2. The FS molecule with an aliphatic chain length of 2.4 nm of the –CH_2_– and –CH_2_CH_2_O– units, provided through bond tunneling pathway [38]; and the delocalized π electrons, which are present in the aromatic ring, triazole, and alkene units, can bridge the redox species and the electrode surface effectively to facilitate the electron transfer. The redox reaction of Ru(NH_3_)_6_^3+^ has been reported to follow the outer-sphere electron transfer mechanism [39,40]. In this case, physical contact between the redox species and the electrode surface is not essential for the electron transfer process since it is favored by the tunneling mechanism. In the case of the FS-SAM in this study, the aromatic and triazole rings with delocalized electrons facilitate the electron transfer of Ru(NH_3_)_6_^3+^, and the aliphatic chains provided the bond tunneling pathway. In contrast, the redox reaction of Fe(CN)_6_^3−^ follows an inner-sphere electron transfer mechanism, where the access of redox species to the electrode surface is necessary for the electron transfer reaction to occur [40,41].

### 2.4. Electrochemical Response of Hydrophobic Dopamine^+^ on the FS-SAM

**Figure 6:**
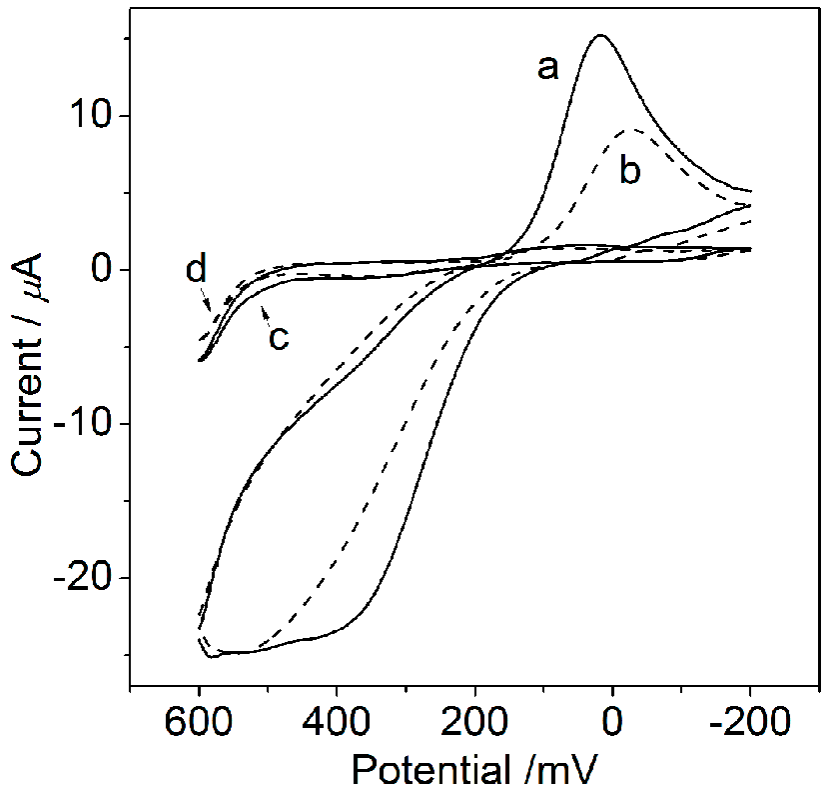
CV of 10 mM DA^+^ on the binary OEG-SAM-modified gold electrodes in 0.1 M KCl (curve **a**) and 0.1 M KPF_6_ (curve **b**), and on the FS-SAM-modified gold electrodes in 0.1 M KCl (curve **c**) and 0.1 M KPF_6_ (curve **d**). pH: 7.0. Scan rate, 100 mV/s. pH: 7.0.

Figure 6 shows CV results for dopamine^+^ (DA^+^) (*p*K_a_ = 8.92) on the binary OEG-SAM and the FS-SAM-modified electrodes. The results are summarized in Table 4. With 0.1 M KCl as a supporting electrolyte, the anodic peak potential (*E_p,a_*) of DA^+^ becomes significantly more positive on the binary OEG-SAM-modified electrode compared with that on the bare gold electrode, as shown in Table 4, and becomes even more positive when we used KPF_6_ instead of KCl, as shown in Table 3 and Figure 4. On the FS monolayer, the hydrophobic, singly charged DA^+^ response nearly disappears.

The slow kinetics and poor responses of DA^+^ on the binary OEG-SAM may result from a large distance of access approach to the monolayer surface, which may be due to unfavorable molecular orientation and interactions of DA^+^ with the monolayer, as well as possible complications in the electron transfer mechanism of DA^+^. The 2e^−^, 2H^+^ redox reaction of DA^+^ shown in Equation (1) may, for example, alter the local pH, affecting surface charge density. Meanwhile, the results reveal that the positive charge of DA^+^ does not significantly facilitate its access to the negative binary OEG-SAM. This phenomenon is different from that of Malem and Mandler’s results [42], which reported improved kinetics of DA^+^ on the negative HS(CH_2_)_2_COO^−^ SAM compared with that of the bare gold electrode. This outcome may result from the short alkyl chain monolayer used in their study.

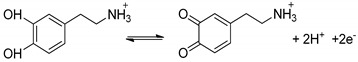
(1)


In previous studies, Chen et al. [12,13] studied the oxidation of different electroactive species at a straight-chain FS-modified gold electrode. The results show that the straight-chain FS-SAM showed an inhibiting effect on the oxidation of glucose, uric acid, ascorbic acid, and oxalate, but facilitated L-cysteine oxidation. They think the low coverage of the adsorbed thiol-containing L-cysteine species might account for the more facile electron-transfer kinetics. They also found that the straight-chain FS-SAM facilitated the electron transfers of tertiary amines oxidation, which may be attributable to the FS adsorption layer being more compact. The amines studied include tri-*n*-ethylamine, tri-*n*-propylamine, and tri-*n*-butylamine. Different from Chen’s results, in our work, the branch-tailed FS-SAM suppressed the electron transfer of dopamine redox. On one hand, it may partly be due to the structural differences between the branch-tailed FS-SAM and the straight-chain ones; on the other hand, it may be caused by the individual differences in the studied species. As we have just investigated the effects of the branch-tailed FS-SAM on only one kind of electroactive organic specie, dopamine, and the obtained data is still very limited. We will conduct further studies on the behavior of more electroactive organic species on the branch-tailed FS-SAMs and compare with the straight-chain ones.

The important feature of surfactant molecules is their amphiphilicity. The specific hydrophobic and hydrophilic portions in the FS-SAM may play a very important role to the response of hydrophobic and hydrophilic probes on the monolayer surface, although other factors may also be important, for example, the electron transfer pathways and electrostatic force. The poor responses of DA^+^ and Fe(CN)_6_^3−^ on the FS monolayer demonstrated that the FS-SAM will suppress the response of both hydrophobic and hydrophilic probes. On one hand, for the hydrophobic DA, the hydrophobic bonding at the hydrophobic FS-SAM exterior and the hydration resistance in the interior may hinder the probe to transport into the film. On the other hand, approaching the hydrophobic exterior is thermodynamically unfavorable for the hydrophilic probes, such as Fe(CN)_6_^3−^. However, as the FS molecule displays a branch structure of the tail group, the exterior is defective thus allowing some degrees of permeation by solvent and the small size hydrophilic electrolyte ions, similar to Cl^−^. Therefore, compared with that in hydrophobic electrolyte solution, the FS-SAM is less resistive in hydrophilic electrolyte solution, displays higher film capacitance, and contributes to faster electrochemical kinetics of electroactive species present in the electrolyte solution.

## 3. Materials and Methods

### 3.1. Materials

Perfluorononene (C_9_F_18_) was obtained from Juhua Group Corporation, Quzhou, China. Azide-derivatized *p*-perfluorononenyloxy benzene (N_3_-Ar-O-C_9_F_17_), ω-tetra (ethylene glycol) hexanethiol (OEG-OH), and ω-propargyl tetra (ethylene glycol) hexanethiol (OEG-CCH) were synthesized in our laboratory (details can be seen in Section 3 in the Supplementary material). Dopamine, KCl, KF, KPF_6_, LiClO_4_, K_3_Fe(CN)_6_, Ru(NH_3_)_6_C1_3_, CuBr, and tris(benzyltriazolylmethyl)amine were purchased from Sigma-Aldrich. The other reagents were commercially available as the highest purity. Doubly distilled deionized water was used for the preparation of the aqueous solutions.

### 3.2. Fabrication of the FS-SAM

The polycrystalline gold electrode (2 mm in diameter, model CHI101, CH Instruments, Inc., Austin, TX, USA) was first polished with chamois leather containing 0.05 μm alumina powders for 10 min, rinsed thoroughly with deionized water, and then was electrochemically cleaned in a 0.5 M H_2_SO_4_ solution by cycling from 0 V to 1.65 V until a stable current profile was observed. After rinsing with deionized water and ethanol, the electrode was dried in a stream of nitrogen, followed by cleaning with H_2_ (3 mbar) plasma in a Harrick Plasma Cleaner (Sterilizer PDG-32G, Ithaca, NY, USA) for 10 min. To form thiols SAM, the cleaned gold electrode was incubated in a mixed 3:1 (molar ratio) OEG-OH/OEG-CCH ethanol solution (total thiol concentration: 0.4 mM) for 24 h, followed by rinsing with ethanol and drying under nitrogen flow. The click reaction was carried out by immersing the propargyl-exposed SAMs in a solution containing 0.1 mM N_3_-Ar-O-C_9_F_17_, 1 mM CuBr, 1 mM l-ascorbic acid sodium salt, and 1 mM tris(benzyltriazolylmethyl)amine in 3:1 dimethyl sulfoxide/water for 30 min. Then, the electrode was taken out and rinsed with ethanol and doubly distilled deionized water.

### 3.3. Surface Characterization

XPS measurements were carried out on a VG Multilab 2000 XPS spectrometer (Thermo Electron Corp., Waltham, MA, USA) using monochromated Al Kα radiation. GIR spectra were recorded on a Bruker Optics Tensor 27 Fourier-transform infrared spectrometer (Ettlingen, Germany) coupled to an external microscope Hyperion 2000 with grazing angle objective (Ettlingen, Germany). The spectral resolution was set at 4 cm^−1^ with 1000 co-addition scans. Water CA experiments were measured on a Dataphysics OCA 20 system (Fielderstadt, Germany) at 25 °C.

### 3.4. Electrochemical Measurements

The electrochemical measurements were performed on an Autolab PGSTAT30 system (Eco Chemie BV, Utrecht, The Netherlands). The conventional three-electrode system used consisted of a saturated calomel reference electrode (SCE), a platinum wire auxiliary electrode, and a gold working electrode. Capacitance (*C*) measurements were conducted by CV scanning in the potential range between 200 and −200 mV vs. SCE at a scan rate of 50 mV/s. The summed cathodic and anodic charging current (*i_c_*) at 0 mV was then divided twice the scan rate and normalized by the electrode area [29]. The EIS data were recorded in the frequency range from 100 kHz to 0.1 Hz, with a root-mean-square amplitude of 10 mV. The Autolab software FRA 4.9.007 was used to fit the impedance data to an appropriate equivalent circuit. From the *R_ct_* values obtained from the impedance plots, the rate constant (*k*_0_) of the electron transfer reactions on the monolayer-modified electrodes were calculated according to the protocol described in References [43,44,45] and the details can be seen in Section 2 in the Supplementary material.

## 4. Conclusions

In this work, we have prepared an amphiphilic branch-tailed FS-SAM on a gold electrode surface via thiol self-assembly followed by a copper (I)-catalyzed “click” reaction modification, and comparatively studied the effect of the FS-SAM system on the electrolyte ion penetration and the electrochemical behavior of redox probes with various hydrophilic/hydrophobic features.

We found that the hydrophilicity/hydrophobicity of the electrolytes exhibit a significant effect on the monolayer capacitance. The FS-SAM displays higher value of the monolayer capacitance in hydrophilic Cl^−^ and F^−^ solutions than that in hydrophobic ClO_4_^−^ and PF_6_^−^ solutions, indicating that the FS-SAM allows higher degree of permeation by the small size hydrophilic electrolyte ions than the large size hydrophobic ones. The high degree of permeation by the electrolytes into the monolayer contributes to fast electrochemical kinetics of electroactive species present in the system.

Moreover, the FS-SAM slowed down the response of both hydrophilic and hydrophobic redox probes, even when the probes carry a charge opposite to that of the SAM surface. For hydrophilic electroactive probes, the redox reaction of the Fe(CN)_6_^3−^ couple is nearly completely blocked by the FS-SAM. By contrast, the electron transfer of Ru(NH_3_)_6_^3+^ is easier than that of Fe(CN)_6_^3−^, which may be partly due to the outer-sphere electron transfer mechanism of Ru(NH_3_)_6_^3+^. The delocalized electrons in the aromatic ring and triazole can favor the electron transfer of Ru(NH_3_)_6_^3+^, and the aliphatic chains can provide a bond tunneling pathway. For hydrophobic DA^+^, the hydrophobic exterior of the FS-SAM does not help its electrochemical response. The hydrophobic bonding between the exterior fluoroalkyl moieties and the hydrophobic probes, as well as the hydration resistance from the interior hydration shell around the OEG moieties, hinders the hydrophobic probes to transport into the FS-SAM. The results may have profound implications for understanding the permselectivity and electron transfers of amphiphilic surfaces consisting of molecules containing aromatic groups and branch-tailed fluorosurfactants in their structures.

## Figures and Tables

**Table 1:** Capacitance (*C*) results of the FS-SAM-modified gold electrodes in different electrolytes.

Electrolyte	0.1 M KCl	0.1 M KF	0.1 M LiClO_4_	0.1 M KPF_6_
*C* (μF/cm^2^)	0.73 ± 0.03	0.55 ± 0.04	0.25 ± 0.01	0.22 ± 0.06

**Table 2:** CV and EIS results for 10 mM Fe (CN)_6_^3−^ on different electrodes.

Probe	Electrode	Electrolyte	Δ*E_p_*/mV	*R_ct_*/Ω cm^2^	*k*_0_/cm·s^−1^
Fe(CN)_6_^3−^	Binary OEG-SAM	0.1M KCl	391	6359	0.42
		0.1 M KPF_6_	602	9374	0.28
	FS-SAM	0.1M KCl	—	15681	0.17
		0.1 M KPF_6_	—	58139	0.05

**Table 3:** CV and EIS results for 10 mM Ru(NH_3_)_6_^3+^ on different electrodes.

Probe	Electrode	Electrolyte	Δ*E_p_*/mV	*R_ct_*/Ω cm^2^	*k*_0_/cm·s^−1^
Ru(NH_3_)_6_^3+^	Binary OEG-SAM	0.1 M KCl	114	1497	1.78
		0.1 M KPF_6_	131	2064	1.29
	FS-SAM	0.1 M KCl	126	1524	1.75
		0.1 M KPF_6_	138	2196	1.22

**Table 4:** CV results for 10 mM dopamine on different monolayer modified gold electrodes.

Electrode	Electrolyte	*E_p,a_*/mV
Bare Au	0.1 M KCl	174
Binary OEG-SAM	0.1 M KCl	347
	0.1 KPF_6_	539
FS-SAM	0.1 M KCl	—
	0.1 KPF_6_	—

## Supplementary Materials

The following are available online, Section 1: Analysis of the composition of the SAMs, Figure S1: Contact angle data and EIS data of the binary OEG-SAMs prepared in the assembly solutions with different OEG-CCH molar fraction, Figure S2: Fractional surface coverage of the SAMs prepared in the assembly solutions with different OEG-CCH molar fraction, Scheme S1: Schematic illustration of the composition of the prepared FS-SAM, Section 2: Detailed computational protocols, Figure S3: Equivalent circuit used for the analysis of EIS data of the electrochemical interface, Section 3: Detailed experimental protocols for the synthesis of compounds, Scheme S2: Synthesis of OEG-OH, Scheme S3: Synthesis of OEG-CCH, Scheme S4: Synthesis of N_3_-Ar-O-C_9_F_17_, Figure S4: ^1^H NMR spectra of the N_3_-Ar-O-C_9_F_17_.
