# Supplementary material for: Electrochemistry Study of Permselectivity and Interfacial Electron Transfers of a Branch-Tailed Fluorosurfactant Self-Assembled Monolayer on Gold
Source: Molecules. 2018 Nov 16;23(11):2998. doi: 10.3390/molecules23112998 (PMC6278534; doi:10.3390/molecules23112998)
Supplement: Supplementary file 1 [file molecules-23-02998-s001.pdf]

Supplementary Material

Electrochemistry study of permselectivity and interfacial electron transfers of a branch-tailed fluorosurfactant self-assembled monolayer on gold

Shanshan Li [1,*], Qingying Luo [1], Zhiqing Zhang [1], Guanghui Shen [1], Hejun Wu [1], Anjun Chen [1], Xingyan Liu [1], Meiliang Li [1] and Aidong Zhang [2,*]

[1] College of Food Science, Sichuan Agricultural University, Ya'an 625014, P. R.China
[2] Key Laboratory of Pesticide & Chemical Biology of Ministry of Education, College of Chemistry, Central China Normal University, Wuhan 430079, P. R. China

* Correspondence: lishanshan.812@163.com; Tel.: +86-155-2700-9697

1 Analysis of the composition of the SAMs

1.1 Analysis of the composition of the binary OEG-SAMs

In order to optimize the composition of the binary OEG-SAM, for further preparing a FS-SAM with a high surface coverage, binary SAMs of alkanethiols with OEG-CCH and OEG-OH in varying fractions were prepared on gold surfaces. After the N_3-Ar-O-C_9F_{17} grafting onto the binary OEG-SAMs, we characterized the obtained FS-SAMs by XPS measurements. The atom fraction (At %) of F in the FS-SAMs are found to be ca. 13.69%, 7.97%, 2.12 % and 2.65%, respectively, when the assembly solutions with OEG-CCH fractions of 25%, 50%, 75% and 100% were used. Obviously, the highest At % of F was obtained on the FS-SAMs prepared from the binary OEG-SAM assembled with 25% OEG-CCH in solution, so the optimal assembly solution for preparing the binary OEG-SAM is with OEG-CCH fraction of 25%. Due to the longer molecule chain of OEG-CCH than that of OEG-OH, in the binary OEG-SAMs, alkynyl groups protruded from the monolayer surface. Meanwhile, OEG-CCH molecules could be separated by OEG-OH, accordingly leading to a space between alkynyl groups. Advantageously, the space contributed to attack of N_3-Ar-O-C_9F_{17} to alkynyl groups on the binary OEG-SAMs, promoting "click" cross-linking reaction. Whereas on the surfaces with more fraction of OEG-CCH, the increased steric hindrance results in the reduction in the efficiency of "click" reaction.

However, the final composition of the binary OEG-SAM on gold is unclear. In previous studies, the composition of a mixed monolayer may be derived by contact angle (CA) [1,2] and EIS measurements [3]. In this work, all these methods were used to solve the compositions of the binary OEG-SAMs, and the results are summarized and compared as a function of the OEG-CCH molar fractions in solution. The CA data are summarized in Figure S1 (A) and the compositional analysis is based on the application of the Cassie [1] and Israelachvili[2] equations. The water CA data on heterogeneous surfaces can be modeled successfully by the Cassie equation. The equation is particularly applicable when the surface is composed of separate, chemically distinct patches. By contrast, when the size of the patches approaches molecular dimensions, the Israelachvili equation is used.

In addition to the water CA data analysis, the EIS data were recorded and are summarized in Figure S1 (B). By referring to Xing's method [3], the composition of the binary OEG-SAMs are analyzed and compared to the fractional surface coverage calculated on the basis of the Cassie and Israelachvili equations, in Figure S2. It can be seen in all cases the OEG-CCH contents are higher in monolayers than in the solutions, indicating a stronger tendency for OEG-CCH to assemble on gold. It is assumed the desolvation effect may play a major contribution to this discrepancy due to the higher solvation of OEG-OH in polar ethanol solvent as compared to OEG-CCH. In general, we observed that the Israelachvili equation describes the compositional data obtained from the analysis of the ESI data better than the Cassie equation. This may indicate that the binary SAMs are mixed on very small length scales [4]. The OEG-CCH fraction in the binary OEG-SAMs is found to be ca. 56%, when the assembly solution with OEG-CCH fractions of 25% was used. When N_3-Ar-O-C_9F_{17} molecules were graft onto the binary OEG-SAMs with OEG-CCH fraction of 56%, the optimum FS-SAM can be obtained, with the highest At % of F at 13.69 %.

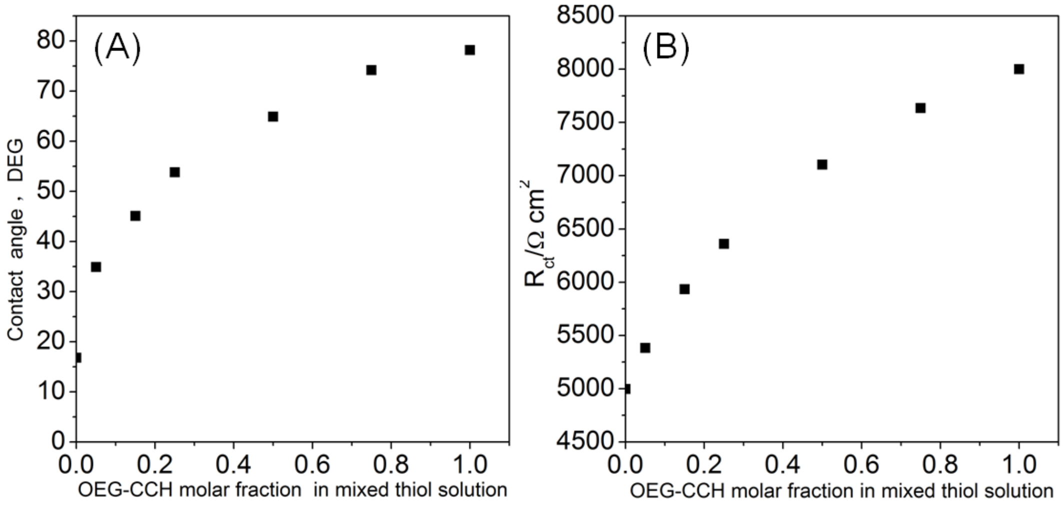

Figure S1. Contact angle data (A) and EIS data (B) of the binary OEG-SAMs prepared in the assembly solutions with different OEG-CCH molar fraction. EIS data was obtained in 0.1 M KCl solution containing 10 mM $K_3Fe(CN)_6$.

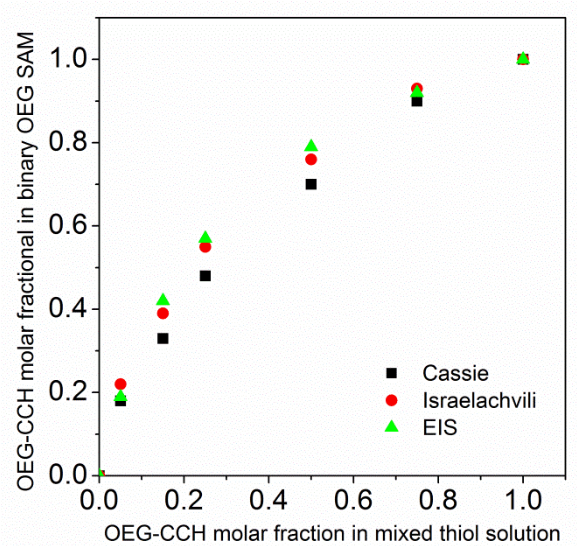

Figure S2. Fractional surface coverage of the SAMs prepared in the assembly solutions with different OEG-CCH molar fraction, as determined from contact angle measurements and the application of the Cassie and Israelachvili equations as well as from EIS data.

1.2 Analysis of the composition of the FS-SAMs

Assuming that all the -CCH crosslinked with the N_3-Ar-O-C_9F_{17}, the average composition of the prepared FS-SAM can be described as $C_{24.08}H_{32.06}F_{9.52}N_{1.68}O_5S$, as shown in Scheme S1. According to that, the theoretical calculated At % of F in the FS - SAM is 12.93%, which is slightly below the measured value 13.69 % by XPS. This may be due to that the -C_9F_{17} groups protruded from the monolayer surface, thus the photoelectron signals of F atoms are more easily detected in XPS measurements than other atoms in the inner layer. Generally, we think that the "click"reaction efficiency is nearly 100%, and the composition of the obtained FS-SAM is 56% FS and 44% OEG-OH.

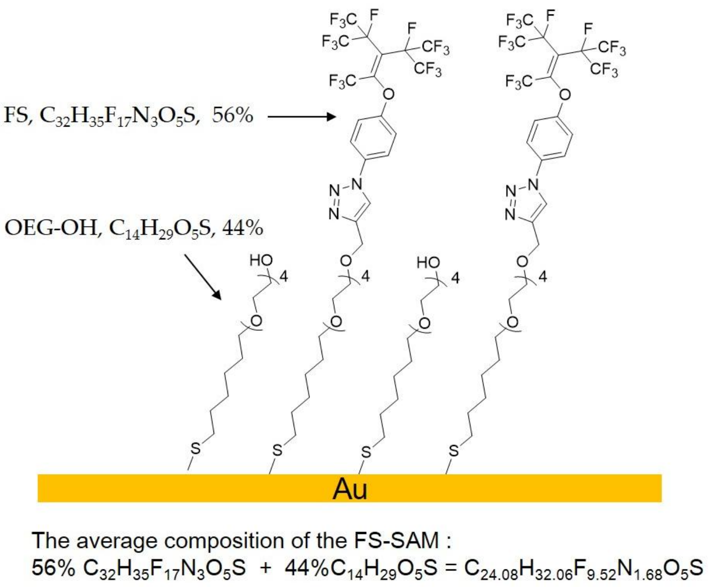

Scheme S1. Schematic illustration of the composition of the prepared FS-SAM.

2 Detailed computational protocols

In the absence of electroactive species, the electrochemical impedance Z of a well-organized SAM covered electrode can be interpreted by a simple equivalent circuit (as shown in Figure S3) of the capacitance in series with the solution resistance (Rs)[5,6] . The total impedance (*Z*) measured in the EIS experiment is

$$Z = R_S + \frac{R_{ct}}{1 + R_W C_T}$$

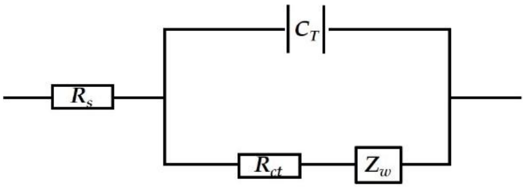

Figure S3. Equivalent circuit used for the analysis of EIS data of the electrochemical interface: (1) R_s, solution resistance; (2) C_T, total capacitance; (3) R_{ct}, charge transfer resistance; (4) R_w, Warburg impedance.

Using the R_{ct} values obtained from the impedance plots, the rate constant value of $Fe(CN)_6^{3-}$ for the binary OEG SAM and FS-SAM modified electrodes were determined as follows [6,7],

$$\theta = 1 - (R_{ct}^{0} / R_{ct})$$

$$k_{app} = RT / n^2 F^2 R_{ct} c$$

$$k_0 = k_{app} / (1 - \theta)$$

where θ is the surface coverage of the monolayers on the gold electrodes, R_{ct}^0 is the charge transfer resistance of bare gold electrode, R_{ct} is the charge transfer resistance of the corresponding monolayer modified electrodes, *R* is the gas constant, *T* is the temperature, *F* is the Faraday's constant, *n* is the number of electrons, *c* is the concentration of the redox couple, k_{app} and k_0 is the apparent and the real rate constants, respectively.

3 Detailed experimental protocols for the synthesis of compounds

3.1 Synthesis of ω-tetra (ethylene glycol)hexanethiol (OEG-OH)

The OEG-OH **4** was synthesized according to the method reported in the literature [8] and the synthesis of PEG-thiol is outlined in Scheme S2. The synthesis was typically performed as follows:

(i) A mixture of 51 mL of tetraethylene glycol **1** (100 mmol), 30 mL THF and 6 mL of 50% NaOH (38 mmol) was heated to 100 °C and stirred for 45 min. Then 9.6 g of 6-bromo-1-hexene (20 mmol) were added to the mixture and refluxed for 16h. The reaction mixture was cooled to room temperature and then 10% HCl solution was added under stirring. The crude product was extracted by ethyl acetate and dried with anhydrous sodium sulfate. Column chromatography (petroleum ether: ethyle acetate, 2:1) afforded pale yellow oily product 2 (31.19 g, yield 88%).

^{1}H NMR (400MHz, $CDCl_3$): δ 5.80 (m, J=15.6Hz 1H, CH_2 =CH-), 4.98 (d, J =10Hz, 2H, CH_2=CH-), 3.70 (m, 16H, -OCH_2CH_2O-), 3.47 (t, J=12.4Hz, 2H,-CH_2CH_2O-), 2.70 (s, 1H,-OH), 2.07 (m, J=20.8Hz, 2H, CH_2= $CHCH_2CH_2$-), 1.60 (m, J=28Hz, 2H,-$CH_2CH_2CH_2O$-), 1.44 (m, J=30.8Hz, 2H, CH_2=$CHCH_2CH_2CH_2$-).

(ii) Under inert atmosphere, a mixture of **2** (0.7g, 300 mmol), 10 mg azobisisobutyronitrile (AIBN), thioacetic acid (0.5 mL, 900 mmol) and 8.4 mL of methanol was UV irradiated at room temperature for 5 h. The mixture was then concentrated in vacuo and purified by column chromatography (elution with petroleum ether: ethyl acetate, 1: 2) to give 3 (0.63g, yield 71%).

^{1}H NMR (400MHz, $CDCl_3$): δ 3.70 (m, 16H, -OCH_2CH_2O-), 3.45 (t, J=11.2Hz, 2H, -CH_2CH_2O-), 2.89 (t, J=12.4Hz, 2H, -CH_2SCO), 2.31 (s, 3H, -$SCOCH_3$), 1.60 (m, 4H, -OCH_2CH_2-, -CH_2CH_2SCO-), 1.38 (m, 4H, -CH_2CH_2-).

(iii) In 50 mL flask, compound **3** (0.5g) was added to 0.1 M HCl (15 mL methanol) was added and the mixture was refluxed at 150 °C for 4h. The mixture was then neutralized by $NaHCO_3$ solution dried with anhydrous sodium sulfate. The product **4** was purified by column chromatography (elution with ethyl acetate) (0.37g, yield 85%).

^{1}H NMR (400 MHz, $CDCl_3$): 3.70 (m, 16H, -OCH_2CH_2O-), 3.45 (t, J= 11.2Hz, 2H, -OCH_2), 2.51 (m, J=7.2Hz, 2H, -SCH_2CH_2-), 1.61 (m, 4H, -OCH_2CH_2-), 1.38 (m, 4H,-CH_2CH_2).

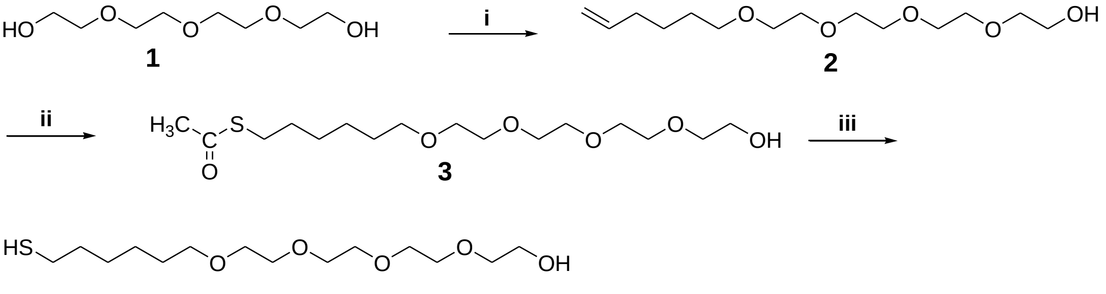

Scheme S2. Synthesis of OEG-OH **4**. ((i) CH_2=$CHCH_2CH_2CH_2CH_2Br$, NaOH solution (50%), 100 °C, 24 h; (ii) CH_3COSH, AIBN, UV light 5h; (iii) 0.1 M HCl in methanol, reflux 4h.)

3.2 Synthesis of ω-propargyltetra(ethylene glycol) hexanethiol (OEG-CCH)

The OEG-CCH **7** was synthesized according to method reported in literature [9]:

(i) The initial synthesis step was coupling of tetraethylene glycol and 6-bromo-1-hexene similar to synthesis of OEG-OH, and the synthesis is outlined in Scheme S3. The next step was the formation of alkyne terminal group. Typically, The synthesized compound **2** (1.38 g, 2.5 mmol) was dissolved in 20 mL of dry CH_2Cl_2. In an ice place 0.30g of NaH (6.25 mmol) was added to compound **2** and 3-bromo-1-propargyl (0.66 g, 2.75 mmol) was slowly added and the mixture was stirred for 30 min. Stirring was continued for 12 hrs at room temperature. The crude product was extracted with ethyl acetate and dried with anhydrous Na_2SO_4. The product **5** was then purified with column chromatography (petroleum ether: ethyl acetate, 4:1) to result in 1.33g, yield 85%.

^{1}H NMR (400MHz, $CDCl_3$): δ 5.78 (m, 1H, J= 15.6Hz, CH2=CH-), 4.98 (d, 2H, J=10Hz, CH_2=CH-), 4.19 (s, 2H, -OCH_2C≡) 3.70(m, 16H, –OCH_2CH_2O-), 3.47 (t, J=12.4Hz, 2H, –CH_2CH_2O-), 2.43 (s, 1H, ≡CH), 2.05 (m, 2H, J=6.4Hz, CH_2=$CHCH_2CH_2$-), 1.60 (m, J=28Hz, 2H,

$-CH_2CH_2CH_2O-$), 1.44 (m, J= 30.8Hz, 2H, $CH_2=CHCH_2CH_2CH_2-$).

(ii) The formation of alkyne terminal group was followed by the modification of terminal double bond to thiol. Typically, a mixture containing 8-10 mg of AIBN, 0.3g of compound **5** and 3.6 mL of methanol was added under inert atmosphere. To the mixture, 0.22 mL thioacetic acid (900 mmol) was added. The mixture was then irradiated with UV lamp under vigorous stirring for 5 hrs. The purification of compound **6** was performed by column chromatography (petroleum ether: ethyl acetate = 3:1) to obtain colorless oily 0.22g, yield about 60%.

^{1}H NMR (400MHz, $CDCl_3$): δ 4.20 (s, 2H, $-OCH_2C≡$), 3.70 (m, 16H, $-OCH_2CH_2O-$), 3.45 (t, J= 11.2Hz, 2H, $-CH_2CH_2O-$), 2.89 (t, J= 12.4Hz, 2H, $-CH_2SCO$), 2.44 (s, 1H, ≡CH), 2.33 (s, 3H, $-SCOCH_3$), 1.60 (m, 4H, $-OCH_2CH_2-$, $-CH_2CH_2SCO-$), 1.38 (m, 4H, $-CH_2CH_2-$).

(iii) Then, at 150 °C, a mixture containing 0.3g of compound **6** and 15 mL 0.1 M HCl in methanol was refluxed for 4 hrs. After the completion of the reaction, saturated $NaHCO_3$ solution was added to neutralize excess HCl. The product **7** was then purified with column chromatography (elution with ethyl acetate) to obtain 0.21g, yield 80%.

^{1}H NMR (400MHz, $CDCl_3$): δ 4.20 (s, 2H, $-OCH_2C≡$), 3.70 (m, 16H, $-OCH_2CH_2O-$), 3.45 (t, J= 11.2Hz, 2H, $-OCH_2$), 2.51 (m, J= 7.2Hz, 2H, $-SCH_2CH_2-$), 2.05 (s, 1H, ≡CH), 1.61 (m, 4H, $-OCH_2CH_2-$), 1.38 (m, 4H, $-CH_2CH_2$).

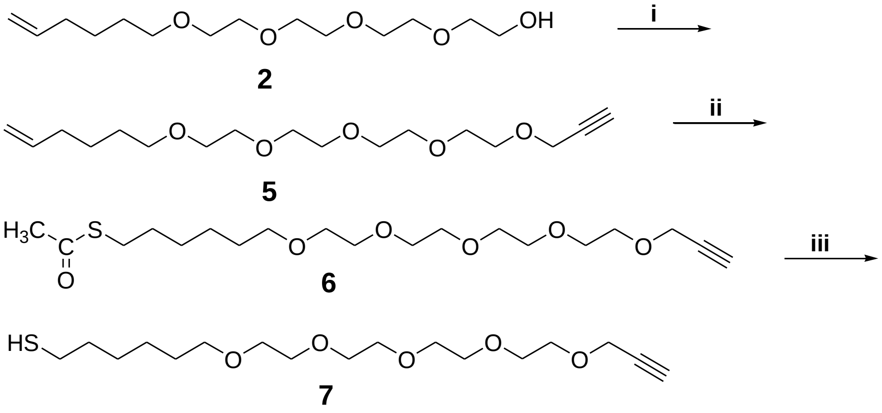

Scheme S3. Synthesis of OEG-CCH **7** ((i) NaH, $BrCH_2C≡CHCH_2Cl_2$, (ii) CH_3COSH, AIBN, light 5 hrs; (iii) 0.1 M HCl in methanol, reflux 4 hrs)

3.3 Synthesis of azide derivatized p-perfluorononenyloxy benzene (N_3-Ar-O-C_9F_{17})

The synthesis of N_3-Ar-O-C_9F_{17} is outlined in Scheme S4:

(i) A mixed solution of 4-(hydroxymethyl) phenol **8** (0.28 g) and sodium azide (0.9 g) in glacial acetic acid (3 mL) was diluted in CH_2Cl_2 (50mL) and stirred at 50 °C for 3h, yielded the azide-terminated product 4-(azidomethyl)phenol **9**.

(ii) The product **9** was purified by column chromatograph. Product **9** (0.2 g) was then introduced into a three-necked flask with the stirrer and reflex condenser. Anhydrous tetrahydrofuran (THF, 30 mL), K_2CO_3 (0.22g) and perfluorononene **10** (0.8 g) were then added into the flask by turns, stirred and reflux at 70 °C for 2h. The obtained oily mixture was washed with distilled water and then with saturated NaCl solution. Finally, the obtained liquid was dried with Na_2SO_4 and then dried under reduced pressure. The obtained product **11** was then purified with column chromatography (petroleum ether: ethyl acetate = 3:1) to obtain 0.26g,

yield 33.3%.

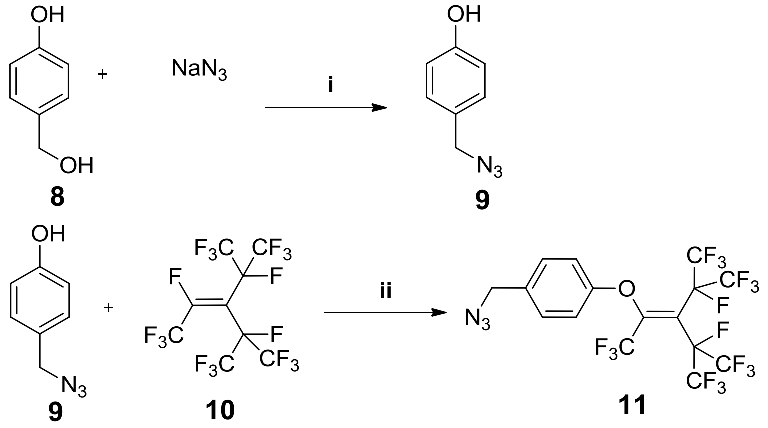

Scheme S4. Synthesis of N_3-Ar-O-C_9F_{17}. ((i) CH_3COOH, 50℃, 3h; (ii) THF, K_2CO_3, 70℃, reflux 2 hrs)

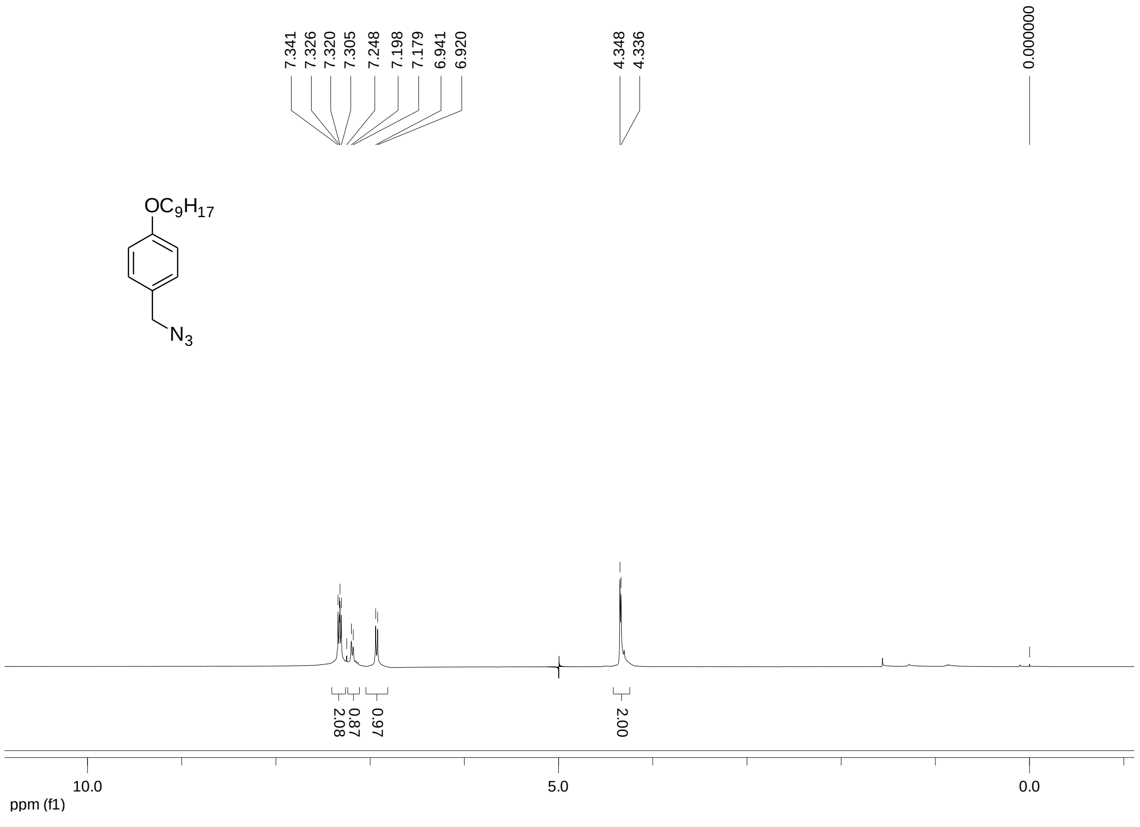

Figure S4. ^{1}H NMR spectra of the N_3-Ar-O-C_9F_{17} (400MHz, $CDCl_3$).

References

1 A. B. D. Cassie, Discussions of the Faraday Society. Faraday Soc, 3 (1948) 11–16.

2 J. N. Israelachvili, M. L. Gee, Contact angles on chemically heterogeneous surfaces, Langmuir, 5(1) (1989) 288-289.

3 Y. F. Xing, S. F. Y. Li, A. K. H. Lau, S. J. O'Shea, Electrochemical impedance spectroscopy study of mixed thiol monolayers on gold, Journal of Electroanalytical Chemistry, 583(2005) 124–132.

4 H. Bayat, D. Tranchida, B. Song, W. Walczyk, E. Sperotto, H. Schönherr, Binary self-assembled monolayers of alkanethiols on gold: Deposition from solution versus microcontact printing and the study of surface nanobubbles. Langmuir, 27(4) (2010) 1353-1358.

5 L. Yang, W. Wei, J. Xia, H. Tao, P. Yang, Electrochemical studies of derivatized thiol self-assembled monolayers on gold electrode in the presence of surfactants. Analytical sciences, 21(6) (2005) 679-684.

6 H. O. Finklea, D. A. Snider, J. Fedyk, E. Sabatani, Y. Gafni, I. Rubinstein, Characterization of octadecanethiol-coated gold electrodes as microarray electrodes by cyclic voltammetry and ac impedance spectroscopy, Langmuir, 9 (1993) 3660-3667.

7 V. Ganesh, S. K. Pal, S. Kumar, V. Lakshminarayanan, Self-assembled monolayers (SAMs) of alkoxycyanobiphenyl thiols on gold-A study of electron transfer reaction using cyclic voltammetry and electrochemical impedance spectroscopy, Journal of colloid and interface science, 296(2006) 195-203.

8 (a) L. Dreesen, Y. Sartenaer, A. Peremans, P. A. Thiry, C. Humbert, J. Grugier, J. Marchand-Brynaert, Synthesis and characterization of aromatic self-assembled monolayers containing methylene and ethyleneglycol entities by means of sum-frequency generation spectroscopy, Thin Solid Films, 500 (2006) 268-277. (b) C. Pale-Grosdemange, E. S. Simon, K. L. Prime, G. M. Whitesides, Formation of self-assembled monolayers by chemisorption of derivatives of oligo (ethylene glycol) of structure HS $(CH_2)_{11}(OCH_2CH_2)_mOH$ on gold, Journal of the American Chemical Society, 113(1) (1991) 12-20.

9 R. Gruškienė, G. Čiuta, R. Makuška, Grafting of poly (ethylene glycol) to chitosan at c (6) position of glucosamine units via "click chemistry" reactions, chemija, 20 (2009) 241-249.
